# ﻿Phelypaeaboissierif.lutea (Orobanchaceae), a peculiar new form from Turkey and typification of the name of this species

**DOI:** 10.3897/phytokeys.186.77575

**Published:** 2021-12-09

**Authors:** Ümit Subaşı, Óscar Sánchez Pedraja, Renata Piwowarczyk

**Affiliations:** 1 Ege University Faculty of Sciences, Department of Biology, 35100, İzmir, Turkey Ege University İzmir Turkey; 2 Grupo Botánico Cantábrico, ES-39722 Liérganes (Cantabria), Spain Grupo Botánico Cantábrico Liérganes Spain; 3 Center for Research and Conservation of Biodiversity, Department of Environmental Biology, Institute of Biology, Jan Kochanowski University, Uniwersytecka 7, PL-25–406 Kielce, Poland Jan Kochanowski University Kielce Poland

**Keywords:** Forma nova, lectotype, Orobanchaceae, *Phelypaea*, SE Turkey, synonyms, typification

## Abstract

The genus *Phelypaea* includes three holoparasite species with one of the most intense red flowers among all plants worldwide. So far, there are few references to other colour taxa of this genus. We describe a new yellow-coloured form, Phelypaeaboissierif.lutea, from Hakkari province in the Cilo Mountains of SE Turkey, found at an altitude of 2,470 m. In typical *P.boissieri*, here typified by us, the flowers are deeply red, and stems, calyx and scales are red to brown, or rarely pale-brown, whereas in the entire population of f.lutea the corolla, calyx, and scales are yellow to orange with black folds in the corolla, while only the stem is brownish.

## ﻿Introduction

The genus *Phelypaea* L. (≡ *Diphelypaea* Nicolson, nom. illeg.) (Orobanchaceae) includes three holoparasite species: *P.coccinea* (M. Bieb.) Poir., *P.boissieri* (Reut.) Stapf, and *P.tournefortii* Desf., whose parasitise Asteraceae hosts. *P.coccinea* occurs in the Caucasus and Crimea, and is a parasite of *Psephellus* Cass. and *Centaurea* L., rarely *Klasea* Cass., while *P.tournefortii* occurs in the Caucasus and Turkey, and is a parasite of *Tanacetum* L. (Sánchez Pedraja et al. 2016; Piwowarczyk et al. 2019). Meanwhile, *P.boissieri* shows a different distribution; it occurs in the Balkans (Albania, Greece, North Macedonia), and Western Asia (Turkey, Iraq and Iran), and parasitises *Centaurea* and occasionally *Cousinia* Cass. in Iraq (Sánchez Pedraja et al. 2016; Piwowarczyk et al. 2019). *P.boissieri* is molecularly, morphologically and regarding host preferences similar to *P.coccinea* (Piwowarczyk et al. 2019, 2021). The morphological features that separate *P.boissieri* and *P.coccinea* are as follows: corolla - tube short and cup-shaped; corolla - lobes broadly obovateorbicular to orbicular, overlapping; anthers - hairy (Stapf 1915; Nicolson 1975; Cullen 2010); however, sometimes apparently intermediate features are also observed (Piwowarczyk et al. 2019). Therefore, further research into the variability of *P.boissieri* and the inclusion of more samples for molecular analysis are required (Piwowarczyk et al. 2021).

Species from the genus *Phelypaea* are achlorophyllous and possess one of the most intense red flowers among all plants worldwide. A recent study on *P.tournefortii* showed that anthocyanins were found in unprecedented large quantities in the flowers, as well as large amounts of polyphenols, especially eukovoside (Piwowarczyk et al. 2020).

So far, there have been few references to colour forms other than red in the genus *Phelypaea* in the literature. One of them is P.coccineaf.aurantiaca Beck in Engl., Pflanzenr. 96: 261 (1930), which was described from Karabakh in the Caucasus based on material collected by Radde as having an orange corolla (“aurantiaco”). Another non-red colour form is *P.helenae* Popl., described from Alushta in Crimea (“corolla orange-yellow”) and, according to Novopokrovsky and Tzvelev (1958: 28), it is synonymous with the species mentioned before and does not constitute a different species (but rather a case of polychromism) with regard to *P.coccinea* (“corolla bright-red”), and both yellow and red forms occur together in Crimea. In *P.coccinea*, sometimes the corolla in the lower-side is orangish or yellow, with the upper side in the typical red color preserved (Piwowarczyk et al. 2019). Within one population, there may be individuals with a corolla which is red on both sides, as well as with one yellow-orange side.

In this paper, we typify *P.boissieri* and indicate its synonyms, and besides, describe a new yellowish-coloured form from Turkey.

### Typification of *Phelypaeaboissieri*

*Phelypaeaboissieri* (Reut.) Stapf in Bull. Misc. Inform. Kew 1915, 6: 291 (1915 [17 Aug 1915]).

**Basionym**: Anoplanthusbiebersteiniivar.boissieri Reut. in DC., Prodr. 11: 42 (1847 [25 Nov 1847]).

**Figure 1.**
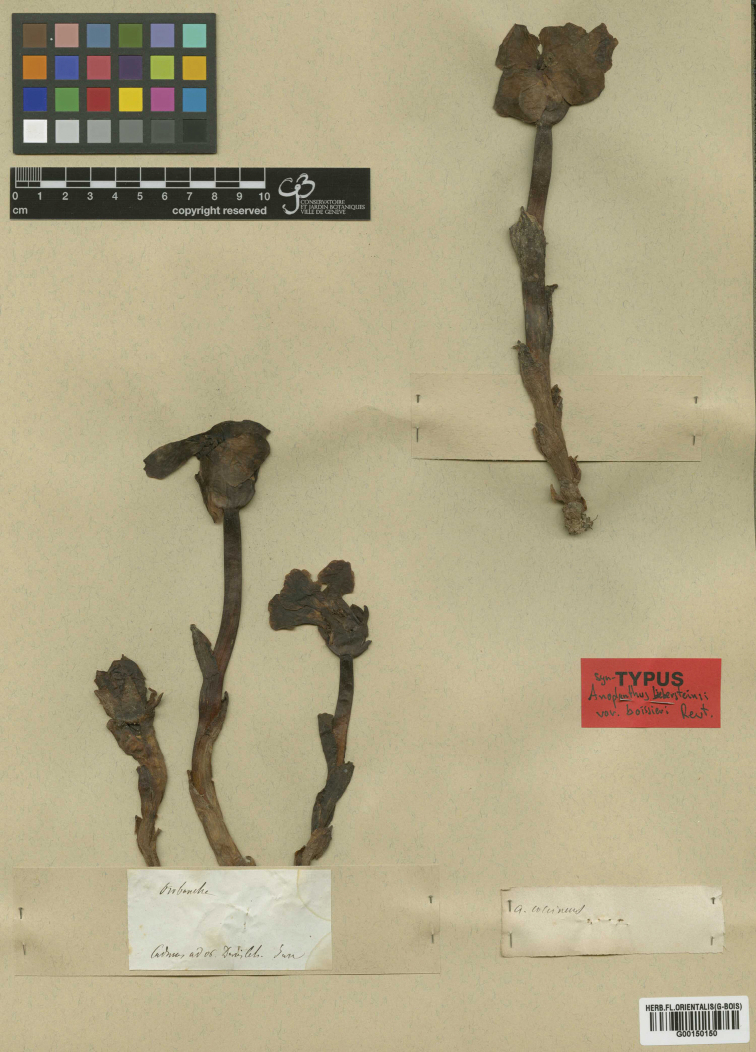
Lectotype and isolectotype (G00150150 - G-Boiss) of *Phelypaeaboissieri*.

**Type**: lectotype (here designated): 1. “*Orobanche* / Cadmus [Mount Cadmus / Topçambaba Dağı / Baba Dağı / Baba Dagh, Aydın Province? or, more likely, mont Honaz / mont Cadmus / Honaz Dağı, Denizli Province?] ad or. Denisleh [to the east Denizli] Jun” [m. Boissier]. – 2. “*A.coccineus*”. – 3. “Syntypes / Anoplanthusbiebersteinii/var.boissieri Reut.” (G-Boiss G00150150 [Fig. 1, the sheet contains four specimens, the lectotype is formed by the three specimens of a single gathering, located in the lower left of the sheet]).

**Isolectotypes**: idem (G-Boiss G00150150 [Fig. 1, the isolectotype is a single specimen in the upper right corner]); “Anoplanthusbiebersteiniivar.boissieri Reut. / Cadmus supra Denisleh [Denizli], ad *Centaurea* / *Boissier*, [6 Jun 1842] (JE00000021 – hb. Haussknecht [two specimens of a single gathering]); “Herb. E. Boissier / *Anoplanthusbiebersteinii* Reut. / β *Boissieri* Reut. / Mesogis [Messogis Mountains / Mesogis Mountains, Aydın Dağları (ancient Lydia), near Aydın / Güzelhisar / Tralles, Aydın Province] et Cadmus [Mount Cadmus / Topçambaba Dağı / Baba Dağı / Baba Dagh, Aydın Province? or mont Honaz / mont Cadmus / Honaz Dağı, Denizli Province?] / Jun. 1842” (P02970934 - Boissier 1842 [the three specimens of a single gathering on the lower right corner close to Boissier’s label]); idem (P02970938 – hb. E. Cosson [the two specimens of a single gathering on the upper left corner close to Boissier’s label]).

**Figure 2.**
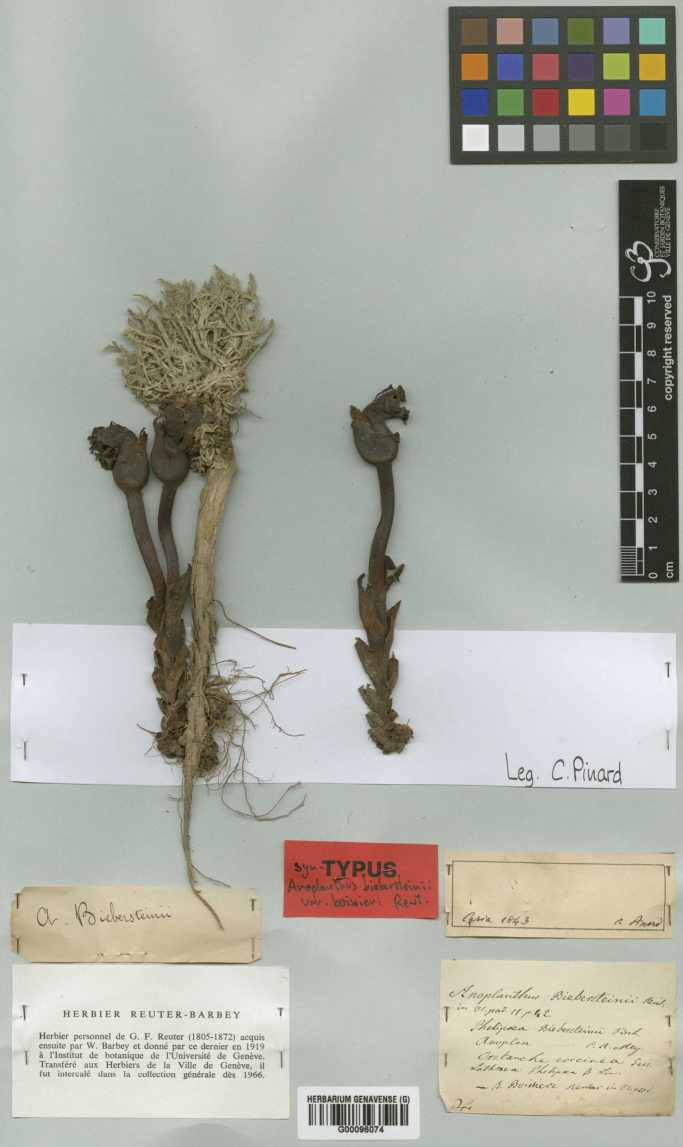
Isosyntype (G00096074 - hb. Reuter-Barbey) of *Phelypaeaboissieri*.

**Isosyntypes**: 1. “Caria [Karia], *C. Pinard*, 1843”. – 2. “Syntypes / Anoplanthusbiebersteinii/var.boissieri Reut.” (G-Boiss G00150149 [two specimens of a single gathering, which are attached to its host-plant]); 1. “Caria [Karia], *C. Pinard*, 1843”. – 2. “*A.biebersteinii*”. – 3. “*Anoplanthusbiebersteinii* Reut. / in DC. prod. 11 p. 42 / *Phelipaeabiebersteinii* Fisch. / *Anoplon* –– C.A. Mey. / *Orobanchecoccinea* Pers. / *Lathraeaphelipaea* β Linn. / –– β. *Boissieri* Reut. in DC. prod.”. – 4. “Syntypes / Anoplanthusbiebersteinii/var.boissieri Reut.” (G00096074 - hb. Reuter-Barbey [Fig. 2, three specimens of a single gathering, two of them are attached to their host-plant])).

**Homotypic synonyms**: *Diphelypaeaboissieri* (Reut.) Nicolson in Taxon, 24 (5–6): 654 (1975), nom. illeg.

Phelypaeacoccineavar.boissieri (Reut.) Beck in Engl., Pflanzenr. 96: 43 (1930)

Diphelypaeacoccineavar.boissieri (Reut.) Uhlich in Kochia 9: 83 (2015), nom. illeg.

**Heterotypic synonyms**: Anoplanthuscoccineusvar.nigrovittatus Boiss., Fl. Orient. 4(2): 494 (1879 [Apr-May 1879]) [“A.coccineus γ nigrovittatus”] (Stapf 1915: 290, 293). *Ind. lo*.: “Hab. in graminosis montis Pir Omar Gudrum [Chiya-i Piromar / Chiyā-i Pīrōmar / Jabal Biru Mar / Jabal Bīrū Mār / Pīr `Umar, Bīrūmā / Piromar / Pīrōmar, Iraqi Kurdistan, Iraq] 5–6000’ (*Haussk* !)”. *Lectotype* (designated here, or perhaps holotype): 1. “711 / *Anoplanthusbiebersteinii* Reuter / fl sanguin., vitta nigra lata / In gramin. 5-6000’ / Haussknecht, It. Orient, Pir Omar Gudrum. Jun. — 1867”. – 2. “*A.coccineus* γ *vittatus*”. – 3. “*Anoplanthuscoccineus* γ *vittatus*” (G-BOIS G00768924 - n° SIB 436114/1 (Stapf 1915: 293) [five specimens of a single gathering]).

Phelypaeacoccineavar.boissierif.septemfida Gilli in Feddes Repert. 46: 47 (1939) [“Calyx irregulariter septemfidus”]. *Ind. loc*.: “Nord-Iran [Iran / Persia]: Nördlich vom Kendewan-Paß [Kendavan Pass / Gardaneh-ye Kandovān, 36°9'0"N, 51°18'0"E, Māzandarān, Alborz / Alburz / Elburz / Elborz Mountains] an einem Abhang unweit des Tunnelbaues, 1800 m; 8.VII.1936.”

The species typified here was described by Reuter, with the rank of var., on the basis of collections made in western Anatolia (Turkey): Mount Cadmus by Boissier and Caria by Pinard (Reuter 1847: 42). Later, Boissier (1879: 494) included it within his *Anoplanthuscoccineus*, which would be the origin of future confusions between the two species. In addition, he described two new varieties, the var.peduncularis (*P.tournefortii* Desf.) and the var.nigrovittatus (*P.boissieri* (Reut.) Stapf). Stapf (1915) raised the variety of Reuter to the rank of species and clarified the differences between the three taxa, so that nowadays we believe that they form this genus. Beck (1930), in a monograph on the family, preferred to continue treating our species with the rank of var. within *P.coccinea*, but created some confusion when giving the distribution of this species where he included the var. described by Reuter. Finally, Cullen (2010) complements the description of *P.boissieri*, giving good illustrations with analysis, providing a new key for the three species, and indicating the distribution of the species treated here.

### A new form of *Phelypaeaboissieri*

#### ﻿ Phelypaea boissieri f. lutea

Taxon classificationPlantaeLamialesOrobanchaceae

Ü. Subasi, R. Piwowarczyk, Ó. Sánchez Pedraja f. nov.

4818A5FC-A271-5768-A343-CAAA7644D870

Figure 3

##### Diagnosis.

**Figure 3.**
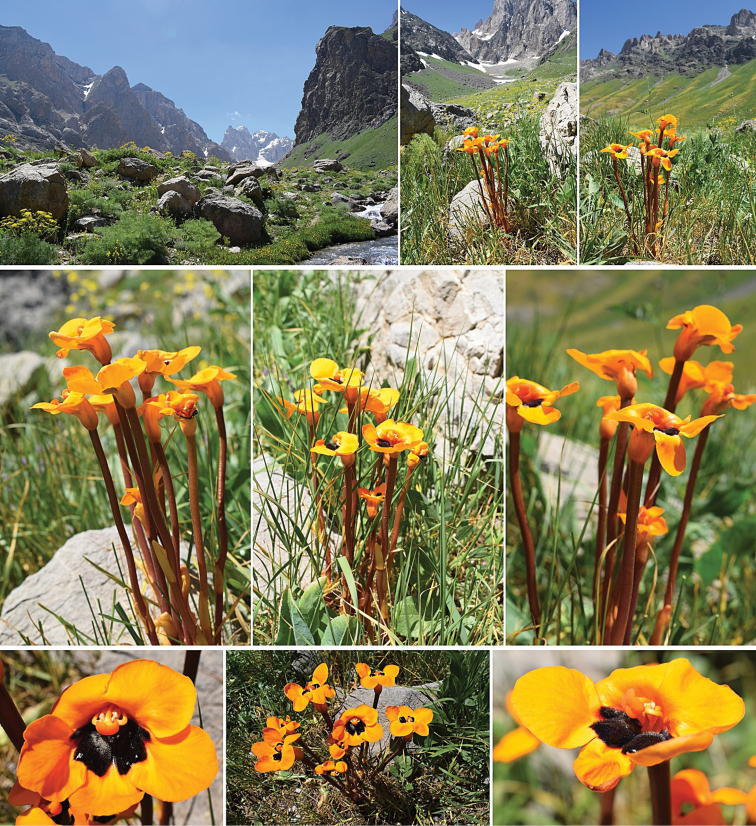
Habitat and general habit of Phelypaeaboissierif.lutea at 2,470 m alt. in Cilo Mountains, Hakkari province, SE Turkey. Phot. Ü. Subaşı.

This new taxon is very similar to typical *Phelypaeaboissieri*, and is compliant with morphometric characteristics in the description of the plant after Reuter (1847: 42, sub Anoplanthusbiebersteiniivar.boissieri), Stapf (1915), Nicolson (1975), Cullen (2010) but differs significantly in color and, usually, a higher stem. In typical *P.boissieri*, flowers are deeply red, and stem, calyx and scales are red to brown, or rarely pale-brown, whereas in the f.lutea corolla, calyx, and scales are yellow to orange with black folds in the corolla, with only the stem being brownish (Fig. 3).

##### Type.

Turkey. C10 Hakkâri: Merkez district, Kırıkdağ village, Gümüşlü location (Kurdish: Spixane). Cilo Mountains, subalpine grasslands and meadows, ca. 2,470 m alt., 10 June 2021, *Ü. Subaşı* s.n. (holotype and isotypes ANK!).

##### Distribution and ecology.

This taxon is known exclusively from the type locality. The Cilo (Glacial) Mountains (Cilo (Buzul) Dağı) are part of the Hakkâri Dağları range and include the third highest peak of Turkey with an altitude of 4,135 meters. Turkey’s Eastern Anatolia Region, the easternmost extension of the Southeast Taurus Mountains, is located within the borders of Hakkari province. The locality of P.boissierif.lutea is located in Hakkari province, Kırıkdağ village, Gümüşlü location (Kurdish: Spixane). This area is located around Mt Uludoruk (Reşko 4,135 m), at an altitude of approximately 2,470 meters. In this area dominated by high mountain steppe plants, the bedrock consists mainly of tectonic deposits and limestone. It is approximately 20 km from any settlements. Parasitising *Centaurea*, probably *C.persica* Boiss. (new host-plant). Phenology - flowering end of June to July, fruiting July to August. The population size is small, over a dozen individuals. The number of individuals counted in the area is fewer than 100. The entire population in this locality is formed by higher plants than the type with yellow corolla. In the future, research into the cause, phytochemical composition, ecological importance and adaptation, and also into pollinators of typical red *Phelypaea* individuals and yellow-orange ones could shed new light on this topic.

##### Etymology.

The form name originated from the yellow to orange colouring of plants.

##### Note.

There are also photos of a yellow flower belonging to *P.boissieri* in Internet sources [https://www.flickr.com/photos/camerar/2887571252/ and http://www.agaclar.net/forum/1296397-post1573.htm]. The photos, respectively, were taken in SE Turkey by Karen Nichols in 26 June 2008, possibly in the same Hakkari province, but no more detailed location data is available, and Hakkari-Mergan-Yaylası (Mergan Plateau) by Güngör Salman in 16 June 2014 [http://www.agaclar.net/forum/1296397-post1573.htm], as well as from Yüksekova/Hakkari by Leoš Smutný in 13 May 2014 [https://www.inaturalist.org/observations/71616618]. We are also aware of a near mention (“Yüksekova [Gever / Gawar], 1950 m, 23 v 1966, *Eiselt*!”) (Gilli 1982: 2, sub *P.coccinea*); this record probably corresponds to the same f. previously described.

## Supplementary Material

XML Treatment for Phelypaea boissieri f. lutea
